# Mitigating Quality Deterioration of Reduced-Fat Pork Sausages During Cold Storage via Resistant Starch Incorporation: Gel Properties and Protein Conformation Study

**DOI:** 10.3390/gels11100763

**Published:** 2025-09-23

**Authors:** Guanghui Liu, Jingchao Fan, Li Wang, Minghui Liang, Chun Xie, Zhuangli Kang

**Affiliations:** 1 School of Pharmacy, Shangqiu Medical College, Shangqiu 476100, China; fanjingchao1979@163.com (J.F.); maomao0492@163.com (L.W.); liangminghui123@126.com (M.L.); xspring9090@163.com (C.X.); 2 College of Tourism and Culinary, Yangzhou University, Yangzhou 225127, China; 008107@yzu.edu.cn

**Keywords:** resistant starch, color, texture characteristics, β-sheet, sausage

## Abstract

This study investigated the changes in pH, water retention, color, texture characteristics, protein conformation, thiobarbituric acid reactive substances (TBARSs), total volatile basic nitrogen (TVB-N), and total plate count in reduced-fat sausages. It explored the quality differences between sausages with and without the addition of resistant starch during storage at 4 °C over a period of 1 to 30 days. The results indicated that TBARS and TVB-N values significantly increased (*p* < 0.05) with the extension of refrigeration time, and the α-helix and β-sheet structures were transformed into β-turn and random coil structures, leading to a significant decrease in the pH, L* and a* values, texture characteristics, and chewiness of all sausages, as well as a significant increase in storage loss and centrifugation loss. Under the same refrigeration time, the sausage with added resistant starch exhibited better water retention and texture characteristics compared to the treatment without resistant starch. Additionally, the TBARS and TVB-N values were significantly lower (*p* < 0.05) in the former. Therefore, the incorporation of resistant starch can effectively slow down the deterioration of gel properties and the increase in total bacterial count in reduced-fat sausages during refrigeration.

## 1. Introduction

Fat is an essential constituent of sausages and plays a crucial role in determining and maintaining their overall quality. During emulsification processing, fat, when combined with muscle proteins and water, forms a stable emulsified system [1,2]. This system enhances the water and fat retention capacity of sausages, thereby improving their flavor and textural properties [3,4]. However, excessive consumption of fat is linked to various health concerns, including hypertension, obesity, and cardiovascular and cerebrovascular diseases [5,6]. Consequently, the sausage industry has increasingly focused on controlling fat content and developing strategies to reduce saturated fatty acid levels.

Resistant starch, a novel type of dietary fiber, cannot be digested or absorbed in the human stomach or small intestine but reaches the colon intact, where it undergoes fermentation by gut microbiota [7,8]. It offers multiple physiological benefits, such as lowering blood glucose and lipid levels, reducing the risk of cardiovascular diseases, and potentially preventing colon cancer [8]. These properties enable resistant starch to be used in managing dyslipidemia and insulin-related disorders, as well as in the development of weight-loss foods. It also holds promise for the dietary management of type 2 diabetes and coronary heart disease [9,10]. Resistant starch can be classified into five types based on structure and source: the physically embedded type (RS1), natural granular type (RS2), regenerated starch type (RS3), chemically modified type (RS4), and composite type (RS5) [8,9]. RS2 can be fermented by intestinal microorganisms in the ileum, lowering luminal pH and facilitating the absorption of minerals such as magnesium, calcium, and zinc through epithelial cells [11]. However, the natural content of resistant starch in most conventional foods is relatively low, limiting its efficacy in regulating blood sugar levels [8,9]. As a functional ingredient, resistant starch contributes to enhancing both the quality and nutritional value of meat products. Wang, Zhou, and Chen [12] demonstrated that incorporating resistant starch into muscle gels improves protein digestibility by reducing the particle size of hydrolysis products, promoting the breakdown of high-molecular-weight proteins, and increasing the release of free amino acids. Furthermore, Wang et al. [13] investigated the effects of four different types of resistant starch on the processing characteristics of low-fat emulsified meat products and found that all types improved water retention, emulsion stability, texture, and color. Our previous research [14] revealed that partially substituting pork back fat with resistant starch, along with a mixture of ice and water, effectively reduces the fat content and caloric value of pork paste products. This substitution also enhances emulsification stability, gel strength, and rheological properties, reduces water mobility, and increases the proportion of immobilized water. Nonetheless, our research was not conducted on the quality changes of such products under refrigerated storage conditions. We hypothesized that RS incorporation would slow physicochemical and microbial deterioration in reduced-fat sausages via water-binding, antioxidant, and protein-structure stabilizing effects. Therefore, this study aims to investigate the changes in water retention, gel properties, protein conformation, total volatile basic nitrogen content, antioxidant activity, and antibacterial properties of fat-reduced sausages supplemented with resistant starch during 30 days of storage at 4 °C.

## 2. Results and Discussion

### 2.1. pH

**Figure 1:**
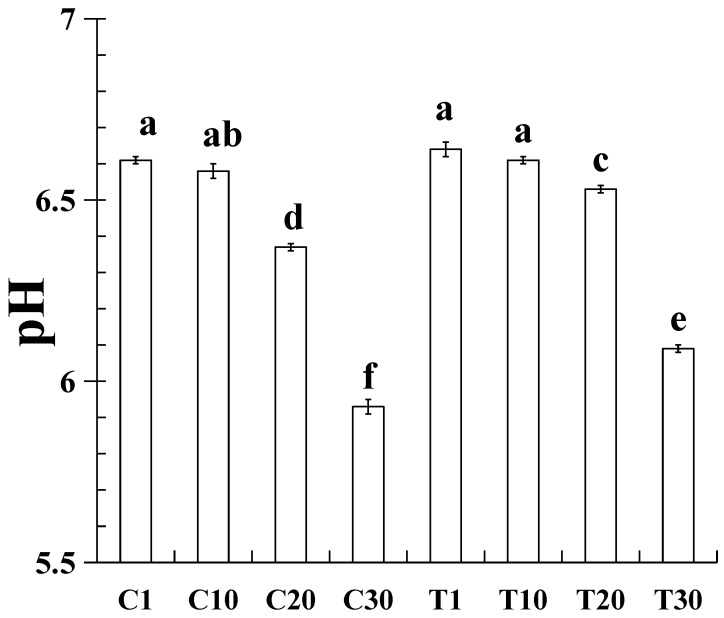
Changes in pH of cooked sausages with pork back fat and/or resistant starch under cold storage. C1, C10, C20, and C30 contained 80 g ice water and 80 g pork back fat and were cold stored on the 1st, 10th, 20th, and 30th days, respectively; T1, T10, T20, and T30 contained 12 g resistant starch, 108 g ice water, and 40 g pork back fat and were cold stored on the 1st, 10th, 20th, and 30th days, respectively. Each value represents the mean ± SE; *n* = 3. ^a–f^ Different parameter superscripts indicate significant differences (*p* < 0.05).

The change in pH is a key indicator reflecting the storage quality of low-temperature sausages, and its dynamic variation is closely associated with microbial activity, chemical denaturation, and processing parameters [15]. As shown in Figure 1, compared to refrigeration for 1 day, there was no significant difference (*p* > 0.05) in pH after 10 days of refrigeration. However, after 20 days of refrigeration, the pH of the reduced-fat sausage with added resistant starch was significantly higher than that of the sausage without resistant starch, indicating that resistant starch can help maintain pH stability during the later stages of refrigeration; the minimum value was 5.93. This decline in pH is primarily attributed to the acidic metabolites produced by microbial metabolism and lipid oxidation [16]. Acid-producing bacteria such as lactic acid bacteria proliferate during refrigeration, converting carbohydrates into organic acids such as lactic acid and acetic acid, which directly lower the pH value [17]. Moreover, during refrigeration, meat proteins gradually degrade into amino acids, leading to an increase in amino groups, while carbohydrates break down into small-molecule organic acids [18,19].

### 2.2. Storage Loss

Sausages may experience exudation of water and fat during refrigeration, which reflects their ability to retain moisture and oil, as well as their overall product quality. As shown in Table 1, the storage loss of sausages significantly increased (*p* < 0.05) with prolonged refrigeration time. This is primarily due to the decline in pH (Figure 1), which compromises the structural integrity of sausages and reduces their water-holding capacity during refrigeration. Additionally, microbial growth during refrigeration can further damage the gel structure of sausages, thereby increasing water and oil loss [20]. Xie et al. [21] reported that cold storage significantly increases the exudation of water and oil from cooked surimi batter. Under the same refrigeration time, the cold storage loss of reduced-fat sausages with resistant starch was lower (*p* < 0.05) than that of those without resistant starch. This observation aligns with the pH trend (Figure 1), where a higher pH corresponds to lower storage loss. Furthermore, resistant starch possesses certain water retention and emulsifying properties [22]. Our previous study demonstrated that the addition of resistant starch reduces the total fluid release, water release, and fat release in raw reduced-fat pork batter while improving cooking yield [14].

### 2.3. Centrifugal Loss

Centrifugal loss is commonly utilized as an indicator to evaluate the texture stability and processing quality of meat products such as sausages and meatballs, which directly influence sensory attributes, including taste, yield, and storage stability [23]. As presented in Table 1, the centrifugal loss of all sausage samples increased significantly (*p* < 0.05) with prolonged refrigeration time. This phenomenon can be attributed to several factors occurring during refrigeration, such as a decrease in pH, microbial proliferation, and the oxidation of proteins and fats. These changes disrupt cross-linking among proteins, resistant starch, fats, and moisture, thereby reducing the water-holding capacity of sausages [24]. Consequently, this leads to increased cold storage loss (Table 1) and elevated centrifugal loss. Previous studies have indicated that during cold storage, the content of free water in cooked surimi batter increases, while the content of immobilized water decreases [21]. The weakened interaction between water molecules and matrix components such as proteins may result in water separation during centrifugation [25]. Under the same refrigeration time, reduced-fat sausages containing added resistant starch exhibited significantly lower centrifugal loss (*p* < 0.05) compared to those without resistant starch. This improvement can be attributed to the enhanced emulsification properties, higher cooking yield, and improved hardness, elasticity, cohesiveness, and chewiness provided by resistant starch [14], which collectively help to mitigate structural degradation during cold storage.

### 2.4. Color

Color changes in sausages during storage are influenced by a combination of factors, including oxidation reactions and microbial growth [26]. Table 2 illustrates the color variations in cooked sausages containing pork back fat and/or resistant starch during cold storage. The L^*^ and a^*^ values decreased significantly (*p* < 0.05) with extended refrigeration time, whereas the b^*^ value increased significantly (*p* < 0.05). One contributing factor is the oxidation of myoglobin upon exposure to atmospheric oxygen, resulting in a gradual darkening of the sausage color. Additionally, oxidation reactions involving substances such as alcohols and aldehydes may further intensify the overall color change [27]. Another factor is microbial proliferation during refrigeration, which consumes nutrients and leads to a loss of original color, resulting in a dull appearance. Moisture loss also plays a critical role in the significant decline (*p* < 0.05) in L^*^ values, as the lack of moisture on the sausage surface reduces light reflectivity [28]. Under identical storage conditions, the a^*^ value of reduced-fat sausages with added resistant starch is significantly lower (*p* < 0.05) than that of sausages without resistant starch. This is likely due to a slight decrease in pH in the former, which reduces the oxidation rate of myoglobin to metmyoglobin, thereby also contributing to the slower increase in b^*^ values [29].

### 2.5. Texture Properties

As shown in Table 3, the hardness of all sausages increased significantly (*p* < 0.05) with prolonged refrigeration time, while springiness and cohesiveness decreased significantly (*p* < 0.05). Chewiness remained relatively stable (*p* > 0.05) during the first 20 days of refrigeration but decreased significantly (*p* < 0.05) after 30 days. Texture changes during refrigeration are primarily associated with factors such as moisture loss, fat oxidation, and structural damage to proteins. For example, enzymes such as calpains and lipases remain partially active at low temperatures and continue to degrade myofibrillar proteins and fats, leading to increased hardness and flavor deterioration [30]. Sausage texture is closely related to moisture content; during refrigeration, moisture loss contributes to an increase in hardness [21]. Additionally, low temperatures may induce fat crystallization, which compromises the gel network and deteriorates texture. Microbial activity can also damage protein structures, further contributing to texture degradation [31]. Under the same refrigeration time, reduced-fat sausages with added resistant starch showed significantly higher hardness, springiness, cohesiveness, and chewiness compared to those without added resistant starch. This suggests that replacing pork back fat with resistant starch can enhance the textural properties of sausages [14]. The significant increase in hardness observed in reduced-fat sausages with added resistant starch during refrigeration may be attributed to the retrogradation of resistant starch at low temperatures, which enhances the firmness of the sausages [8].

### 2.6. Raman Spectroscopy

The conformation changes of sausage proteins during cold storage are closely associated with processes such as protein hydrolysis, oxidation, and structural reorganization [32]. Table 4 presents the alterations in the secondary structure of cooked sausages containing pork back fat and/or resistant starch during refrigeration. The contents of α-helix, β-sheet, and β-turn structures in all sausage formulations exhibited no significant differences (*p* > 0.05) after 1 and 10 days of refrigeration. However, after 20 and 30 days, the levels of α-helix and β-sheet structures significantly decreased (*p* < 0.05), while the β-turn content significantly increased (*p* < 0.05). Additionally, the proportion of random coil structures significantly increased (*p* < 0.05) with prolonged refrigeration time. It is well established that a decrease in pH disrupts hydrogen bonds and electrostatic interactions between protein molecules in sausages, thereby compromising α-helix and β-sheet structures [33]. During refrigeration, free radicals generated from lipid oxidation can attack protein side-chain groups (e.g., thiol and amino groups), leading to protein cross-linking or degradation [34]. It follows from this that it is cooling that leads to the activation of free radicals. While this process is most active, it also involves slight heating. Protein oxidation disrupts intermolecular cross-linking, promoting the transformation of α-helix and β-sheet structures into β-turn and random coil conformations, which in turn reduces water-holding capacity [35]. The β-sheet structure serves as a fundamental component in gel formation; therefore, its reduction contributes to the deterioration of gel texture [36]. Under the same refrigeration time, the sample containing resistant starch in reduced-fat sausages exhibited significantly (*p* < 0.05) higher levels of α-helix and β-sheet structures compared to the group without resistant starch addition. The β-turn content did not show significant variation, whereas the random coil content significantly decreased (*p* < 0.05). The reason for this is that the hydrogen bonding between resistant starch hydroxyl groups and protein side chains is another protective factor [9].

### 2.7. TBARS

The TBARS value is a widely accepted parameter for assessing lipid peroxidation, with an increase indicating elevated oxidative stress [37]. Table 5 illustrates the changes in TBARS values of cooked sausages containing pork back fat and/or resistant starch during cold storage. The TBARS values of all sausage formulations increased significantly (*p* < 0.05) with prolonged refrigeration, except for the T1 and T10 samples, suggesting a progressive intensification of lipid oxidation, reaching a maximum of 0.48 mg MDA/kg. This is primarily due to the susceptibility of unsaturated fatty acids (such as oleic acid and linoleic acid) in sausages to oxidation, which leads to the formation of lipid peroxides that subsequently decompose into aldehydes and other byproducts, thereby increasing TBARS values [38]. The insignificant difference between the T1 and T10 samples can be attributed to the lower fat content in sausages with added resistant starch, resulting in reduced levels of unsaturated fatty acids and, consequently, fewer free radicals. Moreover, resistant starch exhibits free radical scavenging properties, contributing to its antioxidant capacity [39]. Consequently, under the same refrigeration time, the TBARS values of reduced-fat sausages supplemented with resistant starch were significantly lower (*p* < 0.05) than those without resistant starch addition. Furthermore, the decline in pH and changes in the physicochemical properties of sausages may also accelerate the increase in TBARS.

### 2.8. TVB-N

Table 5 presents the variations in TVB-N in cooked sausages containing pork back fat and/or resistant starch during cold storage. TVB-N refers to alkaline nitrogenous compounds, such as ammonia and amines, generated through the enzymatic or microbial degradation of proteins in animal-derived foods. It serves as a key indicator for evaluating the spoilage and deterioration of meat and meat products [40]. In this study, the TVB-N values of all sausage samples increased significantly (*p* < 0.05) with extended refrigeration time, reaching a maximum of 19.36 mg/100 g, which remains below the spoilage threshold of 30 mg/100 g (AOAC (2011) [41]). This is because refrigeration conditions, although inhibitory to spoilage microorganisms, do not completely halt their slow proliferation and subsequent protein degradation [31,42]. Under identical storage conditions, the TVB-N content in reduced-fat sausages with added resistant starch was significantly lower (*p* < 0.05) than in those without starch. For instance, no significant differences were observed between samples C20 and T30. This outcome can be attributed to the superior gel structure of reduced-fat sausages containing resistant starch, which provides excellent water and fat retention properties [14]. Moreover, resistant starch has been shown to inhibit the growth of spoilage bacteria such as *Escherichia coli*, *Enterococcus*, and *Clostridium perfringens*.

### 2.9. Total Plate Count

During the refrigeration process of sausages, the changes in the total number of colonies are influenced by multiple factors such as the composition of raw and auxiliary materials and the initial degree of contamination. As shown in Table 5, the total plate count of all sausages shows an increasing trend with the extension of refrigeration time, and it increases slowly in the early stage of refrigeration and rapidly in the later stage. The main reason for this is that during the initial stage of refrigeration, the original microorganisms in sausages are inhibited or damaged to a certain extent during the sterilization process, but they are not completely killed. Under the new refrigeration environment conditions, these microorganisms adjust themselves to adapt to the new environment [43]. At this stage, the damaged microbial body carries out self-repair and recovery, so the total number of bacteria increase slowly. After a period of self-repair and recovery, the microorganisms adapt to the refrigerated environment and begin to utilize the nutrients in sausages for self-growth and reproduction [44]. Under the same refrigeration time, the total plate count of reduced-fat sausages with resistant starch added was significantly less than that of their non-starch-added counterparts. This is because a good gel structure is not conducive to the growth of microorganisms. In addition, resistant starch can also inhibit the growth of some microorganisms and reduce the total number of colonies in reduced-fat sausages.

## 3. Conclusions

The results indicate that with prolonged refrigeration time, all sausages exhibited a significant increase in storage loss, centrifugal loss, b^*^ values, hardness, TBARS values, and TVB-N values. Conversely, pH, L^*^ and a^*^ values, springiness, and cohesiveness decreased as refrigeration time was extended from 1 to 30 days. Chewiness remained relatively stable during the first 20 days of refrigeration but declined significantly from 19.23 N·mm and 26.14 N·mm to 16.56 N·mm and 23.64 N·mm, respectively, after 30 days. These changes are primarily attributed to protein and fat oxidation, which generate free radicals and compromise the structural integrity of the sausages, leading to a decline in overall quality. The disruption of α-helix and β-sheet structures promotes their transformation into β-turn and random coil structures, with maximum contents of 17.20% and 18.31%, respectively, thereby accelerating gel deterioration. Under the same refrigeration conditions, the variations in pH, storage loss, centrifugal loss, color, hardness, TBARS, TVB-N, and protein secondary structure in reduced-fat sausages with added resistant starch were significantly smaller than those in the control group without resistant starch. This indicates that the addition of resistant starch contributes to the stabilization of sausage quality during refrigeration. In conclusion, incorporating resistant starch enhances water retention capacity, antioxidant activity, and antimicrobial properties, thereby extending the refrigerated shelf life of reduced-fat sausages. This also supports the development of clean-label products and increases consumer acceptance.

## 4. Materials and Methods

### 4.1. Raw Materials

Fresh pork leg meat (4 °C, 24–48 h post-slaughter, pH 5.68 ± 0.02), fresh pork back fat, and food-grade white pepper were obtained from a local slaughterhouse (Shangqiu, China). Both lean meat and pork back fat were separately ground using a 6 mm aperture grinder (RY-22S, Zhengyuan Precision Machinery (Jiangsu) Co., Ltd., Suzhou, China). Subsequently, the meat was vacuum-packed in polyethylene bags and stored at −40 °C for up to two weeks. The fat was ground using a 2 mm aperture plate (RY-22S, Zhengyuan Precision Machinery (Jiangsu) Co., Ltd., Suzhou, China) two hours prior to use. Resistant starch (RS2, 200 mesh, amylose content: 71.50 ± 0.75%) was supplied by the National Starch Company (Westchester, NY, USA). All chemical reagents used were of analytical grade.

### 4.2. Pork Sausage Preparation

Based on a previous study [14], sausages were made using 400 g meat, 7.2 g sodium chloride, and 3 g white pepper. The treatment (C) included 80 g ice water and 80 g pork back fat, while the treatment (T) included 12 g resistant starch, 108 g ice water, and 40 g pork back fat. Briefly, ground meat and sodium chloride were mixed in a meat grinder (Stephan UMC-5C, Hamburg, Germany) at 1500 r/min for 60 s. Half of the ice water was added and chopped for another 60 s, followed by the remaining ice water, pork back fat, and/or resistant starch for 60 s. All chopping was performed in an ice water bath, pausing every 20 s to maintain the pork batter temperature at 4–8 °C. The batter was then stuffed into 26 mm edible collagen casings (Shenguan Holdings (Group) Co., Ltd., Wuzhou, China) using a vacuum stuffer (Shijiazhuang Xiaojin Machinery Manufacturing Technology Co., Ltd., Shijiazhuang, China), linked by hand at 16 cm intervals, weighed, and vacuum-packed in nylon/PE bags. Sausages were cooked in an 80 °C water bath for 20 min (core temperature 72 °C), then stored at 4 °C for 30 days. Samples were taken on the 1st, 10th, 20th, and 30th days. Treatments (C) were labeled C1, C10, C20, and C30, and the other treatments (T) were labeled T1, T10, T20, and T30, respectively.

### 4.3. pH Measurement

The pH of cooked sausage was measured by homogenizing 10 g sausage with 40 mL distilled water at 15,000 rpm for 10 s, using a portable pH meter (PHSJ-4F, Shanghai Yidian Scientiffc Instrument Co., Ltd., Shanghai, China) with automatic temperature compensation.

### 4.4. Storage Loss Measurement

Storage loss was measured on the 1st, 10th, 20th, and 30th days. Sausages were weighed (*W*1), wiped to remove moisture and fat, and reweighed (*W*2). It was calculated as

Cold storage loss (%)=(W1−W2)/W1×100%

### 4.5. Centrifugal Loss Measurement

Cooked sausages were cut into uniform squares, weighed (*W*3), wrapped in filter paper, centrifuged at 5000*× g* for 15 min at 4 °C (Sorvall LYNX4000, Thermo Fisher Scientiffc, Langenselbold, Germany), and reweighed (*W*4). Centrifugal loss was calculated as

Centrifugal loss(%)=(W3−W4)/W3×100%

### 4.6. Color Measurement

The color of the sausage core was measured using a colorimeter (Minolta, Tokyo, Japan) with an aperture of 8 mm, a 10° observer angle, and a D65 illuminant on the 1st, 10th, 20th, and 30th days.

### 4.7. Texture Analysis

Texture was analyzed using a texture analyzer with a P/36R probe (Stable Micro System Ltd., Godalming, UK) [14]. Sausage casings were removed and samples were cut into 15 mm × 15 mm cylinders. The test speed was 2.0 mm/s with 50% strain.

### 4.8. Raman Spectroscopy Measurment

Raman spectroscopy of cooked sausage was performed using the method of Zhu et al. [45] to analyze changes in α-helix, β-sheet, β-turn, and random coil structures.

### 4.9. TBARS Measurement

TBARS was measured using the method of Ulu [46].

### 4.10. TVB-N Measurement

Total volatile basic nitrogen was measured according to the method of AOAC (2011) [41].

### 4.11. Total Plate Count Measurement

Total plate count was measured according to the method of AOAC (2011) [41].

### 4.12. Statistical Analysis

Each experiment was repeated three times using different materials. Data were expressed as mean ± SE. One-way ANOVA and GLM were performed using SPSS v.26.0. Differences were considered significant at *p* < 0.05 using Duncan’s Multiple Range Test.

## Figures and Tables

**Table 1:** Changes in storage loss (%) and centrifugal loss (%) of cooked sausages with pork back fat and/or resistant starch under cold storage.

Sample	Storage Loss (%)	Centrifugal Loss (%)
C1	0 ^f^	8.35 ± 0.37 ^e^
C10	2.26 ± 0.17 ^d^	10.26 ± 0.30 ^d^
C20	5.04 ± 0.29 ^b^	13.07 ± 0.33 ^b^
C30	7.18 ± 0.25 ^a^	14.96 ± 0.26 ^a^
T1	0 ^f^	6.62 ± 0.28 ^f^
T10	1.16 ± 0.19 ^e^	8.30 ± 0.38 ^e^
T20	3.25 ± 0.35 ^c^	9.78 ± 0.29 ^d^
T30	5.37 ± 0.28 ^b^	11.65 ± 0.37 ^c^

C1, C10, C20, and C30 contained 80 g ice water and 80 g pork back fat and were cold stored on the 1st, 10th, 20th, and 30th days, respectively; T1, T10, T20, and T30 contained 12 g resistant starch, 108 g ice water, and 40 g pork back fat and were cold stored at the 1st, 10th, 20th, and 30th days, respectively. Each value represents the mean ± SE; *n* = 3. ^a–f^ Different parameter superscripts indicate significant differences (*p* < 0.05).

**Table 2:** Changes in color of cooked sausages with pork back fat and/or resistant starch under cold storage.

Sample	L^*^ Value	a^*^ Value	b^*^ Value
C1	77.52 ± 0.76 ^c^	1.65 ± 0.25 ^a^	7.35 ± 0.31 ^d^
C10	73.38 ± 0.70 ^e^	1.13 ± 0.21 ^b^	9.32 ± 0.38 ^c^
C20	70.62 ± 0.64 ^f^	0.55 ± 0.23 ^c^	12.16 ± 0.25 ^b^
C30	68.18 ± 0.73 ^g^	0.13 ± 0.18 ^d^	14.33 ± 0.40 ^a^
T1	82.61 ± 0.71 ^a^	1.82 ± 0.21 ^a^	8.63 ± 0.29 ^c^
T10	80.53 ± 0.67 ^b^	1.71 ± 0.25 ^a^	9.25 ± 0.21 ^c^
T20	77.86 ± 0.57 ^c^	1.02 ± 0.20 ^b^	11.46 ± 0.37 ^b^
T30	75.49 ± 0.73 ^d^	0.44 ± 0.22 ^c^	13.98 ± 0.34 ^a^

C1, C10, C20, and C30 contained 80 g ice water and 80 g pork back fat and were cold stored on the 1st, 10th, 20th, and 30th days, respectively; T1, T10, T20, and T30 contained 12 g resistant starch, 108 g ice water, and 40 g pork back fat and were cold stored on the 1st, 10th, 20th, and 30th days, respectively. Each value represents the mean ± SE; *n* = 3. ^a–g^ Different parameter superscripts indicate significant differences (*p* < 0.05).

**Table 3:** Changes in texture properties of cooked sausages with pork back fat and/or resistant starch under cold storage.

Sample	Hardness (N)	Springiness	Cohesiveness	Chewiness (N.mm)
C1	53.39 ± 0.82 ^g^	0.802 ± 0.005 ^c^	0.453 ± 0.009 ^d^	19.44 ± 0.39 ^d^
C10	56.35 ± 0.76 ^f^	0.782 ± 0.008 ^d^	0.436 ± 0.006 ^e^	19.23 ± 0.37 ^d^
C20	59.51 ± 0.79 ^e^	0.757 ± 0.006 ^e^	0.408 ± 0.008 ^f^	18.46 ± 0.33 ^de^
C30	63.07 ± 0.91 ^c^	0.708 ± 0.005 ^f^	0.371 ± 0.010 ^g^	16.56 ± 0.29 ^f^
T1	58.73 ± 0.66 ^e^	0.866 ± 0.007 ^a^	0.534 ± 0.012 ^a^	27.16 ± 0.41 ^a^
T10	61.05 ± 0.73 ^d^	0.837 ± 0.005 ^b^	0.511 ± 0.009 ^b^	26.14 ± 0.27 ^a^
T20	65.26 ± 0.56 ^b^	0.810 ± 0.006 ^c^	0.487 ± 0.011 ^c^	25.70 ± 0.30 ^ab^
T30	68.77 ± 0.84 ^a^	0.788 ± 0.009 ^d^	0.451 ± 0.008 ^d^	23.64 ± 0.35 ^c^

C1, C10, C20, and C30 contained 80 g ice water and 80 g pork back fat and were cold stored on the 1st, 10th, 20th, and 30th days, respectively; T1, T10, T20, and T30 contained 12 g resistant starch, 108 g ice water, and 40 g pork back fat and were cold stored on the 1st, 10th, 20th, and 30th days, respectively. Each value represents the mean ± SE; *n* = 3. ^a–g^ Different parameter superscripts indicate significant differences (*p* < 0.05).

**Table 4:** Changes in protein secondary structure (α-helix, β-sheet, β-turn, and random coil, %) of cooked sausages with pork back fat and/or resistant starch under cold storage.

Sample (%)	α-Helice	β-Sheet	β-Turn	Random Coil
C1	57.72 ± 2.27 ^a^	17.32 ± 1.38 ^c^	13.76 ± 0.36 ^c^	11.45 ± 0.20 ^e^
C10	56.09 ± 1.68 ^a^	16.41 ± 1.26 ^c^	13.85 ± 0.29 ^c^	13.63 ± 0.18 ^c^
C20	52.83 ± 2.33 ^b^	14.28 ± 1.37 ^d^	15.04 ± 0.32 ^b^	17.79 ± 0.23 ^b^
C30	49.02 ± 1.82 ^c^	12.11 ± 1.41 ^de^	16.73 ± 0.31 ^a^	21.93 ± 0.22 ^a^
T1	53.21 ± 1.79 ^b^	21.63 ± 1.77 ^a^	14.12 ± 0.28 ^c^	11.13 ± 0.23 ^e^
T10	52.11 ± 2.42 ^b^	21.05 ± 1.36 ^a^	14.36 ± 0.26 ^c^	12.53 ± 0.19 ^d^
T20	50.29 ± 1.90 ^bc^	19.80 ± 1.62 ^b^	15.77 ± 0.30 ^b^	14.22 ± 0.19 ^c^
T30	48.43 ± 1.96 ^d^	16.23 ± 1.55 ^c^	17.20 ± 0.33 ^a^	18.31 ± 0.22 ^b^

C1, C10, C20, and C30 contained 80 g ice water and 80 g pork back fat and were cold stored on the 1st, 10th, 20th, and 30th days, respectively; T1, T10, T20, and T30 contained 12 g resistant starch, 108 g ice water, and 40 g pork back fat and were cold stored on the 1st, 10th, 20th, and 30th days, respectively. Each value represents the mean ± SE; *n* = 3. ^a–e^ Different parameter superscripts indicate significant differences (*p* < 0.05).

**Table 5:** Changes in TBARS (mg MDA/kg), TVB-N (mg/100 g), and total plate count (CFU/g) of cooked sausages with pork back fat and/or resistant starch under cold storage.

Sample	TBARS (mg MDA/kg)	TVB-N (mg/100 g)	Total Plate Count (CFU/g)
C1	0.291 ± 0.007 ^e^	2.79 ± 0.28 ^f^	4.00 × 10
C10	0.322 ± 0.005 ^d^	6.67 ± 0.25 ^d^	3.20 × 10^2^
C20	0.371 ± 0.006 ^b^	11.65 ± 0.29 ^b^	2.13 × 10^3^
C30	0.480 ± 0.007 ^a^	19.39 ± 0.31 ^a^	9.62 × 10^3^
T1	0.213 ± 0.005 ^f^	2.02 ± 0.20 ^g^	5.00 × 10
T10	0.227 ± 0.008 ^f^	4.46 ± 0.29 ^e^	1.10 × 10^2^
T20	0.285 ± 0.007 ^e^	8.90 ± 0.26 ^c^	7.90 × 10^2^
T30	0.352 ± 0.008 ^c^	12.17 ± 0.22 ^b^	3.68 × 10^3^

C1, C10, C20, and C30 contained 80 g ice water and 80 g pork back fat and were cold stored on the 1st, 10th, 20th, and 30th days, respectively; T1, T10, T20, and T30 contained 12 g resistant starch, 108 g ice water, and 40 g pork back fat and were cold stored on the 1st, 10th, 20th, and 30th days, respectively. Each value represents the mean ± SE; *n* = 3. ^a–g^ Different parameter superscripts indicate significant differences (*p* < 0.05).

## Data Availability

The original contributions presented in this study are included in the article. Further inquiries can be directed to the corresponding author.

## References

[1] Zhang J., Li D., Zhang Y., Tang J., Shi S., Zeng X., Chen H., Pang J., Wu C. (2025). The effects of soy protein isolate-based composite gels as pork back fat substitutes in low-fat emulsified sausage. Food Res. Int..

[2] Kang Z., Xie J., Li Y., Song W., Ma H. (2023). Effects of pre-emulsified safflower oil with magnetic field resistant soy 11S globulin on the gel, rheological, and sensory properties of reduced-animal fat pork batter. Meat Sci..

[3] Honrado A., Aínsa A., Marquina P.L., Beltrán J.A., Calanche J.B. (2022). Low-fat fresh sausage from rabbit meat: An alternative to traditional rabbit consumption. Meat Sci..

[4] Jin G., Zhang M., Wang X., Zhang Y., Jiang G., Mei L. (2025). Characteristics of exopolysaccharides—Egg white protein composite gel and its application in low—Fat sausage. Food Chem. X.

[5] Christensen J.J. (2024). From puzzle pieces to picture: Genetic data helps refine our understanding of the link between liver fat and heart disease risk. Atherosclerosis.

[6] Pirola L., Ciesielski O., Balcerczyk A. (2022). Fat not so bad? The role of ketone bodies and ketogenic diet in the treatment of endothelial dysfunction and hypertension. Biochem. Pharmacol..

[7] Bojarczuk A., Khaneghah A.M., MarszaLek K. (2022). Health benefits of resistant starch: A review of the literature. J. Funct. Foods.

[8] Hu H., Jiang H., Sang S., McClements D., Jiang L., Wen J., Jin Z., Qiu C. (2024). Research advances in origin, applications, and interactions of resistant starch: Utilization for creation of healthier functional food products. Trends Food Sci. Technol..

[9] Raigond P., Ezekiel R., Raigond B. (2015). Resistant starch in food: A review. J. Sci. Food. Agr..

[10] Das M., Santra S., Chakraborty M., Rajan N., Sarvanabhupathy S., Anusha, Biswas P., Banerjee R. (2024). Resistant starch: Insights into better health and metabolism. Bio. Agr. Biotech..

[11] Metzler-Zebeli B.U., Canibe N., Montagne L., Freire J., Bosi P., Prates J.A.M., Tanghe S., Trevisi P. (2019). Resistant starch reduces large intestinal pH and promotes fecal lactobacilli and bifidobacteria in pigs. Animal.

[12] Wang X., Zhou Z., Chen C. (2022). In vitro digestion of a mixed gel of pork muscle and resistant starch: Salt-soluble protein perspective. Food Chem..

[13] Wang X.X., Li S.M., Wang J., Bao K.X., Zhou Z.K. (2025). Comparative effects of four types of resistant starch on the techno-functional properties of low-fat meat emulsions. Food Chem..

[14] Xie C., Liu G.H., Liang M.H., Li S.H., Kang Z.L. (2024). Applying Resistant Starch to Improve the Gel and Water Retention of Reduced-Fat Pork Batter. Gels.

[15] Zhang L., Zhang M., Mujumdar A.S., Yu D., Wang H. (2023). Potential nano bacteriostatic agents to be used in meat-based foods processing and storage: A critical review. Trends. Food Sci. Technol..

[16] Zhuang H., Li X., Wu S., Zhao J., Gao Y., Yan H. (2022). Application of ginseng powder and combined starter culture for improving the oxidative stability, microbial safety and quality characteristics of sausages. LWT Food Sci. Technol..

[17] Yang Q., Feng Z., Yuan Y., Xia X., Liu Q., Chen Q., Kong B. (2025). Unraveling the potential of zinc protoporphyrin-forming lactic acid bacteria for replacing nitrite and their role in quality characteristics of Harbin dry sausage. Food Chem. X.

[18] Günal-Köroğlu D., Yılmaz H., Subasi B.G., Capanoglu E. (2025). Protein oxidation: The effect of different preservation methods or phenolic additives during chilled and frozen storage of meat/meat products. Food Res. Int..

[19] Ehsani A., Hashemi M., Afshari A., Aminzare M., Raeisi M., Tayebeh Z. (2020). Effect of different types of active biodegradable films containing lactoperoxidase system or sage essential oil on the shelf life of fish burger during refrigerated storage. LWT Food Sci. Technol..

[20] Lin X.X., Liu C.S., Cai L., Yang J.R., Zhou J.C., Jiang H.Z., Shi Y.H., Gu Z.F. (2021). Effect of High Hydrostatic Pressure Processing on Biochemical Characteristics, Bacterial Counts, and Color of the Red Claw Crayfish *Cherax quadricarinatus*. J. Shellfish Res..

[21] Fan J.C., Liu G.H., Wang K., Xie C., Kang Z.L. (2023). Effects of Potassium Bicarbonate on Gel, Antioxidant and Water Distribution of Reduced-Phosphate Silver Carp Surimi Batter under Cold Storage. Gels.

[22] Ou Y., Chen K., Guo J., Ye C., Zhou X., Chen D., Li B., Liu C., Liu J. (2025). Advances in resistant starch: Mechanisms, applications, and challenges in obesity management and low-fat food development. Int. J. Bio. Macromol..

[23] Zhang M., Mittal G.S., Barbut S. (1995). Effects of test conditions on the water holding capacity of meat by a centrifugal method. LWT-Food Sci. Technol..

[24] Liu R., Du X., Li Y., Zhou J., Xia X. (2025). Research progress on the formation, influencing factors, and mitigation strategies of advanced glycation end products in meat-based foods during frozen storage and processing. Trends Food Sci. Technol..

[25] Zhao X., Guo R., Li X., Wang X., Zeng L., Wen X., Huang Q. (2023). Effect of oil-modified crosslinked starch as a new fat replacer on gel properties, water distribution, and microstructures of pork meat batter. Food Chem..

[26] Feng C., Makino Y. (2020). Colour analysis in sausages stuffed in modified casings with different storage days using hyperspectral imaging—A feasibility study. Food Con..

[27] Sellimi S., Ksouda G., Benslima A., Nasri R., Rinaudo M., Nasri M., Hajji M. (2017). Enhancing colour and oxidative stabilities of reduced-nitrite turkey meat sausages during refrigerated storage using fucoxanthin purified from the Tunisian seaweed *Cystoseira barbata*. Food Chem. Toxicol..

[28] Kang Z.L., Yao P.L., Zhao S.M., Hou Q., Xu J.G., Ma H.J. (2024). Effect of temperature and sodium bicarbonate combined on aggregation, rheology and conformation of low-salt chicken myofibrillar protein. LWT-Food Sci. Technol..

[29] Xie C., Shi B., Liu G., Li S., Kang Z. (2023). Using Potassium Bicarbonate to Improve the Water-Holding Capacity, Gel and Rheology Characteristics Reduced-Phosphate Silver Carp Batters. Molecules.

[30] Uysal C., Enişte İ., Çifçi M., Şimşek A., Kılıç B. (2022). Effects of different packaging methods and storage temperatures on physicochemical, microbiological, textural and sensorial properties of emulsion-type sausage chips. J. Stored Products Res..

[31] Wang P., Xu X., Zhou G. (2009). Effects of Meat and Phosphate Level on Water-Holding Capacity and Texture of Emulsion-Type Sausage During Storage. Agric. Sci. China.

[32] Sun Y., Tang H., Zou X., Meng G., Wu N. (2022). Raman spectroscopy for food quality assurance and safety monitoring: A review. Cur. Opin. Food Sci..

[33] Yang K., Zhou Y., Guo J., Feng X., Wang X., Wang L., Ma J., Sun W. (2020). Low frequency magnetic field plus high pH promote the quality of pork myofibrillar protein gel: A novel study combined with low field NMR and Raman spectroscopy. Food Chem..

[34] Chen Q., Xie Y., Yu H., Guo Y., Yao W. (2023). Non-destructive prediction of colour and water-related properties of frozen/thawed beef meat by Raman spectroscopy coupled multivariate calibration. Food Chem..

[35] Moudache M., Nerín C., Colon M., Zaidi F. (2017). Antioxidant effect of an innovative active plastic film containing olive leaves extract on fresh pork meat and its evaluation by Raman spectroscopy. Food Chem..

[36] Li Y.P., Zhang X.H., Lu F., Kang Z.L. (2021). Effect of sodium bicarbonate and sodium chloride on aggregation and conformation of pork myofibrillar protein. Food Chem..

[37] Zheng B.Y., Li X.Y., Hao J., Xu D.X. (2003). Meat systems produced with Monascus pigment water-in-oil-in-water multiple emulsion as pork fat replacers. Food Chem..

[38] Feng Y., Zhang Y., Huang K., Li S., Cao H., Guan X. (2025). Multidimensional exploration of the interactions and mechanisms between fat and salt: Saltiness perception, and taste perception and oxidation of fat. Trends. Food Sci. Technol..

[39] dos Santos J.M., Ignácio E.O., Bis-Souza C.V., da Silva-Barretto A.C. (2021). Performance of reduced fat-reduced salt fermented sausage with added microcrystalline cellulose, resistant starch and oat fiber using the simplex design. Meat Sci..

[40] Ozogul F., Polat A., Ozogul Y. (2004). The effects of modified atmosphere packaging and vacuum packaging on chemical, sensory and microbiological changes of sardines (*Sardina pilchardus*). Food Chem..

[41] AOAC (2011). AOAC Official Methods of Analysis of AOAC International.

[42] Ren Q., Fang K., Yang X., Han J. (2022). Ensuring the quality of meat in cold chain logistics: A comprehensive review. Trends. Food Sci. Technol..

[43] Solo-de-Zaldívar B., Tovar C.A., Borderías A.J., Herranz B. (2015). Pasteurization and chilled storage of restructured fish muscle products based on glucomannan gelation. Food Hydrocolloilds.

[44] Jung Y., Oh S., Lee S., Lee H., Choo H., Jo C., Nam K., Lee J., Jang A. (2025). Characterization of meat quality, storage stability, flavor-related compounds, and their relationship in Korean Woorimatdag No. 2 chicken breast meat during cold storage. Poult. Sci..

[45] Zhu D.Y., Kang Z.L., Ma H.J., Xu X.L., Zhou G.H. (2018). Effect of sodium chloride or sodium bicarbonate in the chicken batters: A physico-chemical and Raman spectroscopy study. Food Hydrocolloids.

[46] Ulu H. (2004). Evaluating of three 2-thiobarbituric acid methods for the measurement of lipid oxidation in various meats and meat products. Meat Sci..

